# Host proteases: key regulators in viral infection and therapeutic targeting

**DOI:** 10.3389/fimmu.2025.1671173

**Published:** 2025-09-18

**Authors:** Qiongrong Xia, Xiaohua Liu, Huacui Huang

**Affiliations:** Department of Medical Laboratory, Xindu District People’s Hospital of Chengdu, Chengdu, Sichuan, China

**Keywords:** host proteases, viral infection, immune response, regulatory mechanism, broad-spectrum antiviral agents

## Abstract

Viral infections pose a major threat to global health, causing severe pneumonia, meningitis, hepatitis, and fatal complications. Viruses are highly dependent on host cellular factors to complete their life cycle, and host proteases, as one of the core regulatory hubs, profoundly influence the progression of infection and pathogenicity. Viruses rely on specific host proteases (e.g., transmembrane serine proteases [TMPRSS family], furin, cathepsins, and others such as caspases and metalloproteases) to precisely cleave and activate viral surface glycoproteins and internal precursor proteins, thereby facilitating efficient invasion, replication, release, and immune evasion. Meanwhile, host proteases participate bidirectionally in immune regulation. They can be exploited by viruses to exacerbate pathological damage (e.g., triggering cytokine storms), yet also act as key defense components by directly cleaving viral proteins to inhibit infection. Different viruses have evolved sophisticated strategies to hijack host proteases, whose activity, specificity, and tissue distribution directly determine the viral tissue tropism and pathogenic potential. Compared to highly mutable viral targets, host proteases serve as ideal targets for developing host-directed antiviral drugs (HADs) due to their genetic stability and conserved mechanisms, but their toxicity requires careful evaluation because of their physiological roles. Inhibitor strategies targeting host proteases have demonstrated promising breakthrough potential in circumventing drug resistance and exerting broad-spectrum inhibitory activity against diverse viruses. Elucidating the multidimensional roles of host proteases in infection is crucial for designing the next-generation of broad-spectrum, anti-drug resistance antiviral strategies. This review systematically summarizes the regulatory mechanisms of host proteases at various stages of viral infection and advances in targeted intervention strategies, providing theoretical support for the development of resistance-resistant and broad-spectrum antiviral therapeutics.

## 1 Introduction

Viral infectious diseases persistently threaten global public health, with over 200 viruses known to cause human diseases, yet currently approved antiviral drugs effectively target only approximately 10 viral pathogens (1). Over the past decade, the frequency of emerging disease outbreaks has increased. From the Ebola virus (EBOV) epidemic to the coronavirus disease 2019 (COVID-19) pandemic, these events reveal the severe impact of viral infections. Chronic infections such as human immunodeficiency virus (HIV), hepatitis B virus (HBV), and hepatitis C virus (HCV) have cumulatively affected over 350 million people and claimed more than 40 million lives to date (2, 3). Meanwhile, acute pathogens such as influenza virus (FluV) and severe acute respiratory syndrome coronavirus 2 (SARS-CoV-2) infect over 1 billion people annually, leading to more than 5 million severe cases and over 10 million related deaths (4, 5). Current viral protease-targeting antiviral therapies face two major challenges. First, the issue of drug resistance targeting viral proteases is becoming increasingly prominent. The treatment failure rate of HIV-1 protease inhibitors (e.g., lopinavir) has reached 20% (6, 7), oseltamivir resistance rates in circulating hemagglutinin 1 neuraminidase 1 (H1N1) strains reached 3.76% (8), and the Glu166Val (E166V) mutation in SARS-CoV-2 main protease (M^pro^, also known as 3CL^pro^) reduces nirmatrelvir activity by 100-fold, often causing treatment failure (9–11). Second, narrow-spectrum activity limits efficacy against highly variable viruses with multiple serotypes, such as dengue virus (DENV) (1). These critical challenges urgently require innovative antiviral strategies and targets.

Viruses depend entirely on host cells to accomplish critical life cycle steps, including invasion, replication, maturation, and release. During this process, host proteases, as key enzymes responsible for protein degradation and modification, serve as pivotal regulators at every infection stage, positioning them as high-potential breakthrough targets. Their core value is first evidenced by conserved cleavage mechanisms across viral families. Furin mediates the cleavage of HIV envelope glycoprotein gp160 into gp120/gp41 subunits to initiate membrane fusion (12). It also hydrolyzes human papillomavirus (HPV) late protein 2 (L2) at Arg470, enabling viral genome translocation across the nuclear membrane (13). Such protease-mediated conformational rearrangements are essential for cross-family viral infections. This compartmentalization ensures precise activation of viral proteins at critical subcellular sites. EBOV requires endosomal cathepsins B/L (CTSB/L) under low-pH conditions to trim glycoprotein 1 (GP1) subunits and expose the receptor-binding domain (14–17), while coronaviruses (CoVs) undergo furin-mediated pre-cleavage of spike (S) proteins in the Golgi compartments to enhance infectivity (18, 19). Compartment-specific proteases (e.g., transmembrane protease serine 2 (TMPRSS2)) spatiotemporally optimize cleavage (20, 21). In addition to acting as viral cofactors, host proteases further influence viral proliferation and transmission by modulating host cell signaling (22, 23), immune responses (24, 25), and apoptosis (26, 27). Consequently, targeting host proteases not only blocks the viral life cycle but also pioneers innovative therapeutic strategies by regulating host responses.

Despite the substantial potential of host proteases as antiviral targets, their intrinsic biological properties pose significant challenges for targeted interventions. The same protease often exhibits functional pleiotropy in distinct infection contexts. For example, furin enhances viral particle maturation in HIV infection (28, 29), yet generates incompletely cleaved immature particles in DENV infection, facilitating host cell invasion via non-canonical pathways and exacerbating severe disease (30). Similarly, TMPRSS2 drives respiratory viral infections through its high expression in the bronchial epithelium (31), yet in prostate tissue, it is androgen-regulated and participates in epithelial differentiation and tissue remodeling. Aberrant TMPRSS2 expression may promote prostatic hyperplasia or carcinoma (31). These complexities necessitate intervention strategies with precisely regulated spatiotemporal specificities. A deeper layer of challenge stems from the essential physiological functions performed by these proteases themselves. Taking furin as an example, this critical precursor protein convertase participates in multiple core physiological processes. It regulates neuronal activity by processing β-nerve growth factor (β-NGF) (32); within the skeletal system, it is responsible for the maturation of the pro-hormone pro-osteocalcin (pro-OCN), modulating its activation and endocrine function, while also influencing the secretion of osteoblast-derived metabolic hormones (33). Similarly, TMPRSS2 plays a pivotal role across multiple tissues. In the kidneys, it participates in processing the epithelial sodium channel (ENaC) to regulate sodium reabsorption (34, 35). In the prostate, it is highly expressed and regulates prostatic fluid secretion and sperm function (36). And in the intestine, it maintains intestinal barrier integrity by cleaving the tight junction protein occluding (34). Collectively, these complex biological characteristics underscore that antiviral strategies targeting host proteases must establish a delicate balance between antiviral efficacy and physiological safety. Any intervention crucially requires precise avoidance of interference with their normal physiological functions.

This review focuses on the multifaceted roles of host proteases in viral infections, systematically outlining their core functions across four key stages. These stages include viral entry activation, replication and assembly, release and dissemination, and immune evasion. It also provides an in-depth assessment of innovative intervention strategies targeting these proteases. By analyzing the multiple functions of these proteases, this review aims to pioneer novel therapeutic approaches to suppress viral transmission and pathogenicity and provide a new perspective and theoretical basis for future viral therapeutic research.

## 2 Classification and function of host proteases

Host proteases serve as key effector molecules in virus-host interactions, exerting multifaceted regulatory roles via the viral life cycle through specific peptide bond hydrolysis. Their primary functions include (1) cleavage and activation of viral precursor proteins (e.g., glycoproteins, polyproteins) to confer infectivity or replication capacity; (2) modulation of host signaling pathways to alter cellular states for viral replication or immune evasion; and (3) remodeling of the extracellular matrix or membrane structures to create a favorable microenvironment for viral entry, assembly, or dissemination. Specifically, viruses have evolved a three-pronged strategy to manipulate this system: (i) targeted exploitation of tissue- or cell-specifically expressed protease isoforms [e.g., TMPRSS2 is highly expressed in the respiratory epithelium (37)]; (ii) induction of aberrant protease expression or activation in the infection microenvironment[(e.g., furin cleaves influenza virus HA0 (38, 39)]; (iii) evolution of specific cleavage motifs in viral proteins that are recognized by host proteases [e.g., HIV gp120 contains an REKR cleavage motif (40)]. Based on catalytic mechanisms, host proteases are classified into three major classes (serine proteases, cysteine proteases, and metalloproteases), in addition to aspartic proteases and threonine proteases. Their functional diversity originates from evolutionary divergence in active-site residues and substrate-binding pocket architectures.

### 2.1 Serine proteases

**Figure 1:**
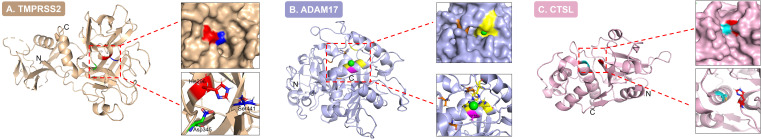
Three-dimensional structures and catalytic center characteristics of three types of core host proteases. **(A)**. In the TMPRSS2 structure (serine protease; PDB: 7MEQ), key residues of the catalytic triad—His296 (red), Asp345 (green), and Ser441 (blue)—are positioned at the active site, demonstrating the conserved catalytic architecture characteristic of serine proteases. **(B)**. The ADAM17 structure (metalloprotease; PDB: 9O54) reveals the catalytic Zn²^+^ ion (green sphere) coordinated by residues Glu406 (magenta), Met435, Ala439, and Leu348 (orange), illustrating the metal ion-dependent catalytic mechanism. **(C)**. Within the CTSL structure (cysteine protease; PDB: 7W34), the catalytic dyad residues Cys25 (blue) and His163 (red) localize to the active pocket, underpinning the catalytic functionality of cysteine proteases.

Serine proteases are characterized by a highly conserved Ser-His-Asp catalytic triad (41), as exemplified by TMPRSS2 (**Figure 1A**). The structural basis for their functional divergence lies in the diverse stereoconformations of substrate-binding pockets. For instance, the catalytic groove of TMPRSS2 precisely accommodates the receptor-binding domain (RBD) of coronavirus spike proteins (e.g., human coronavirus HKU1 (HCoV-HKU1)), inducing conformational changes that trigger membrane fusion (42). Conversely, furin recognizes multibasic cleavage motifs (e.g., Arg-X-X-Arg↓) within the polyproteins of diverse pathogens through its distinctive substrate-binding cleft, mediating their maturation and infectivity (40). During viral infections, serine proteases are extensively exploited to activate both surface glycoproteins (e.g., influenza HA, CoVs S, and HIV gp160) and internal precursor polyproteins—serving as critical rate-limiting steps in viral entry and maturation.

### 2.2 Metalloproteases

Metalloproteases are characterized by an active center coordinated through specific metal ions (e.g., Zn²^+^) coordinated by surrounding amino acid residues (e.g., histidine, glutamate, or aspartate) (43), as exemplified by a disintegrin and metalloproteinase 17 (ADAM17) (**Figure 1B**). Their functional diversity arises from the structural plasticity of the metal-binding and catalytic domains. For example, matrix metalloprotease-2 (MMP-2) and matrix metalloprotease-9 (MMP-9) exemplify this diversity, utilizing catalytic domains to degrade collagen networks with high efficiency (44), while ADAM17 precisely cleaves transmembrane signaling molecules using its zinc-finger motif (45). Under physiological conditions, metalloproteases maintain homeostasis by regulating extracellular matrix remodeling, growth factor release, and the migration of inflammatory cells. Viruses disrupt this balance through dual mechanisms: (1) inducing aberrant overexpression of metalloproteases in the inflammatory microenvironment, such as HBV infection upregulating MMP-9 to promote liver fibrosis (46), or (2) hijacking proteolytic functions to facilitate dissemination routes, as evidenced by HIV exploiting metalloprotease-mediated degradation of extracellular matrix components to enhance cell-to-cell spread (47).

### 2.3 Cysteine proteases

Cysteine proteases feature a cysteine residue at their active site, functioning through a Cys-His catalytic dyad (48), exemplified by cathepsin L (CTSL) (**Figure 1C**). Their activity is tightly regulated by subcellular localization (e.g., pH, redox status). For instance, cathepsin B (CTSB) and CTSL activate viral fusion proteins in the acidic environment of endosomes (15), whereas caspase-3 executes apoptotic cascades in the cytoplasm (49). These enzymes dominate critical host processes—including lysosomal antigen processing, irreversible initiation of programmed cell death, and dynamic cytoskeletal remodeling—all precisely driven by the redox-sensitive thiol activity of their cysteine residues. Viruses hijack these mechanisms through spatiotemporally precise strategies. In the acidic endosomal microenvironment, activated CTSB/CTSL promotes viral entry [e.g., EBOV (14–17)]; in the cytoplasm, they regulate apoptosis-related proteases such as caspases to inhibit or promote cell death, creating a metabolic environment conducive to viral replication [e.g., adenovirus delays apoptosis by inhibiting caspase-3 activity (27)]. The mechanisms by which these microenvironmental molecular interactions determine infection outcomes will be dissected in subsequent mechanistic investigations.

In addition to the above, the host protease network also includes aspartic proteases and threonine proteases. Aspartic proteases utilize an active center formed by two conserved aspartic acid residues (Asp) (50), which enables the specific recognition and cleavage of viral polyprotein substrates in acidic microenvironments. For example, cathepsin D (CTSD) mediates the conformational rearrangement of HIV gp120, thereby facilitating direct interaction with coreceptors and enhancing the efficiency of viral membrane fusion (51). Threonine proteases employ threonine residues as nucleophilic attack centers to drive proteolytic cascades (52). The threonine hydrolase activity of the proteasome plays dual regulatory roles in the rotavirus life cycle, mediating viral capsid uncoating for genome release during invasion, and optimizing viral particle maturation through degradation of host restriction factors during assembly (53, 54). Through substrate-specific cleavage, these two protease classes cooperatively regulate key nodes of the viral replication cycle alongside previously described proteases.

From a molecular evolutionary perspective, the functional diversity of host proteases reflects a dynamic equilibrium forged through protracted virus-host coevolution. For example, the acquisition of furin cleavage sites (e.g., *PRRA* insertion in SARS-CoV-2) by coronavirus spike proteins likely reflects the adaptive evolution of viruses to exploit furin, which is highly expressed in the respiratory mucosa. In response, hosts have evolved defense mechanisms, such as the serine protease inhibitor (serpin) family, to continuously counteract the viral hijacking of proteases. This persistent evolutionary pressure drives the structural plasticity of protease substrate-binding domains, enabling specific viral families to utilize distinct protease subtypes to complete their life cycles, while also shaping the complexity of host defense networks (elaborated in Section 3) (**Table 1**).

**Table 1:** Roles of host proteases in the viral life cycle.

Classification	Protease	Target/Function	Viral life cycle stage	Inhibitors	References
Serine Proteases	TMPRSS2	Cleave Fluv HA	Invasion	Nafamostat, N-0385, Nafamostat mesylate	(20, 58–62, 68, 172–175)
		Cleave CoVs S	Invasion		(20, 21, 82–84)
		As the invasion receptor for HKU1	Invasion		(101–103)
		Activate EBOV GP	Invasion		(106)
	Furin	Cleave Fluv HA	Assembly	Dec-RVKR-cmk, Cypermethrin, MI-1851, luteolin	(55, 83, 137, 186, 187, 189)
		Cleave CoVs S	Invasion		(18, 19)
		Cleave HIV gp160	Invasion		(12)
		Cleave the EBOV GP precursor	Invasion		(16)
		Cleave HPV L2	Invasion		(118)
		Cleave CHIKV E2-E1	Invasion		(126)
		Cleave CCHV PreGn	Assembly		(135, 136)
		Cleave RSV F	Assembly		(137)
		Cleave DENV prM	Assembly		(30)
		Cleave BDV GP	Release		(143)
		Cleave PRV gB	Intercellular transmission		(144, 145)
	HAT (TMPRSS11D)	Cleave Fluv HA	Invasion	Compound 15	(59, 60, 63, 68, 178)
		Weakly activates the cell fusion of CoVs	Invasion		(91)
	TMPRSS4	Cleave Fluv HA	Invasion	–	(64, 65, 68)
	TMPRSS11A	Cleave Fluv HA	Invasion	–	(66)
	Matriptase	Cleave Fluv HA	Invasion	–	(65, 67–70)
	TMPRSS13 (MSPL)	Cleave Fluv HA	Invasion	N-0430	(71, 194)
		Cleave CoVs S	Invasion		(91, 92)
	hepsin	Cleave Fluv HA	Invasion	–	(71)
	trypsin	Cleave HAstV-8 ORF2	Assembly	–	(134)
	prostasin	Cleave Fluv HA	Invasion	–	(71)
	plasmin	Cleave Fluv HA	Invasion	Basic phenylalanine analogs	(73, 74, 179)
	KLK5/12	Cleave Fluv HA	Invasion	–	(75–77)
	Tryptase Clara	Recognizes the Gln-X-Arg motif to activate viruses	Invasion	–	(79, 80)
	TL2	Recognizes the Gln-X-Arg motif to activate viruses	Invasion	–	(79, 80)
		Cleave HIV gp120	Assembly		(79)
	DESC1	Weakly activates the cell fusion of CoVs	Invasion	–	(91)
	Thrombin	Activate HIV gp120	Invasion	–	(104)
		Cleave HEV ORF1	Replication		(129)
	FXa	Cleave CoVs S	Invasion	–	(89)
		Blocks the binding of CoVs spike to ACE2 (under thrombotic pathological conditions).	Invasion		(88)
	ACOT2	Cleave DENV NS2B-NS3^pro^	Replication	–	(128)
Cysteine Protease	CTSL	Cleave CoVs S	Invasion	E64d, Z-FY-CHO, K11777, Adamantane	(20, 83, 85–87, 127, 180, 181)
		Activate EBOV GP	Invasion		(15, 16, 106)
		Cleave RV σ3	Uncoating		(121)
		Cleave HEV ORF2	Invasion		(127)
	CTSB	Synergize with CTSL to activate EBOV GP	Invasion	–	(15)
		Cleave RV σ3	Uncoating		(121)
	CTSS	Cleave RV σ3	Uncoating	–	(120)
	Caspase-6	Cleave lamin A/C	Invasion	Z-VEID-FMK	(124, 195)
		Cleave CSFV NS5A	Replication		(130)
	Caspase-3	Cleave nuclear lamin	Invasion	–	(125)
		Cleave AD2/12 E1A	Replication		(27)
		Cleave ADV NS1	Replication		(131)
		Cleave ARVs mμNS	Release		(147)
Metalloproteas	ADAM17	Mediates the shedding of ACE2	Invasion	–	(95–97)
	AD1M15	Recombinant virus replication organelles	Replication	–	(142)
	TRABD2A	Degrade HIV Gag	Inhibit assembly	–	(139)
	MMP-9	Degrade extracellular matrix components	Intercellular transmission.	–	(47)
Other	elastase	Cleave RSV F	Invasion	–	(107)
	proteinase 3	Cleave RSV F	Invasion	–	(107)

“-” represents “unidentified”.

## 3 Core mechanistic roles of host proteases in viral infection

### 3.1 Host protease regulatory networks in viral invasion

The process of viral invasion into host cells constitutes a dynamic interplay between pathogens and the host protease system. Conformational activation of viral surface glycoproteins, driven by host protease-mediated site-specific proteolytic cleavage, constitutes the essential initial step in the infection cascade. This regulatory strategy exhibits remarkable viral specificity and evolutionary adaptability.

#### 3.1.1 Host protease-dependent activation mechanisms of influenza viruses

**Figure 2:**
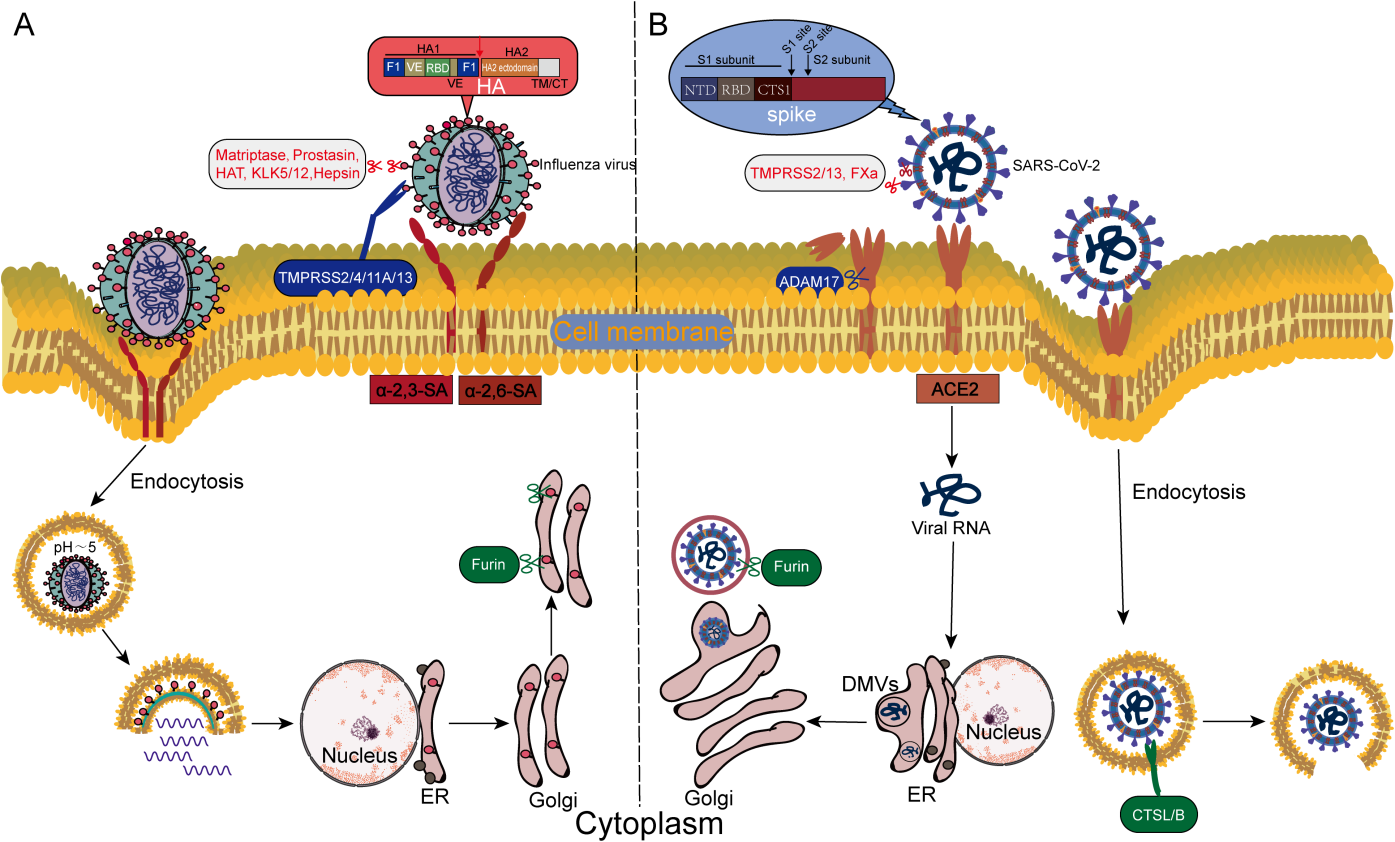
Differences in the host protease-dependent invasion mechanisms of influenza virus and SARS-CoV-2. **(A)** Influenza virus invades via the endosome-dependent pathway: Host proteases (e.g., TMPRSS2, cathepsins) cleave the HA, exposing the fusion peptide, which mediates the fusion of the viral envelope with the endosomal membrane in the acidic endosomal environment. **(B)** SARS-CoV-2 employs a dual-pathway strategy: On the plasma membrane surface, furin, TMPRSS2/13, and FXa cleave the S1/S2 or S2’ sites of the spike protein (S), directly triggering immediate fusion between the viral envelope and the host cell membrane; In the endosomal pathway, CTSL/B cleave the S protein, driving fusion between the viral envelope and the endosomal membrane.

The influenza virus hemagglutinin (HA) glycoprotein functions as the principal mediator of viral entry, and its proteolytic processing determines infection efficiency, exhibiting significant subcellular localization specificity and viral subtype dependency. The HA precursor (HA0) must be cleaved by host proteases to generate HA1/HA2 subunits, thereby exposing the N-terminal fusion peptide (GLFGAIAGFIE) and enabling the virus to acquire membrane fusion capability—it is particularly emphasized that although uncleaved HA0 can bind to sialic acid receptors on host cells, it completely loses the ability to drive membrane fusion (38, 39, 55, 56). This critical cleavage event occurs at two stages of the viral life cycle. Furin primarily cleaves HA0 (especially in highly pathogenic H5/H7 subtypes) in the host cell’s Golgi apparatus during viral assembly and release, allowing newly formed virions to acquire fusion potential before release (55, 57); in contrast, TMPRSS2 and others complete the cleavage on the surface of host cell membranes during the viral entry stage (58–62). This activation process is orchestrated through cooperative actions within the host protease network, including human airway trypsin-like protease (HAT, also referred to as TMPRSS11D) (59, 60, 63), TMPRSS2 (58–62), TMPRSS4 (64, 65), TMPRSS11A (66), and matriptase (*ST14* gene) (65, 67–70) (**Figure 2A**), which catalyze HA cleavage on the cell membrane surface to induce fusogenic conformational changes, thereby facilitating infection across diverse influenza subtypes. Notably, influenza subtypes exhibit distinct protease dependencies. TMPRSS2 is indispensable for HA activation of H7N9 and H1N1pdm in primary human bronchial epithelial cells, consistent with its high expression in respiratory mucosal epithelia. In contrast, TMPRSS4 dominates HA processing of H3N2 and influenza B viruses in murine alveolar type II epithelial cells due to its specific distribution in lung parenchymal cells (37, 71). In the absence of TMPRSS2, TMPRSS13 (alias MSPL, matriptase-like protease), hepsin, and prostasin maintain viral infectivity through compensatory cleavage (71). This segregation of protease function likely reflects the divergent expression profiles of type II transmembrane serine protease (TTSP) family members.

Influenza C virus (ICV) employs a distinct mechanism, wherein its hemagglutinin-esterase (HE) fusion protein, which has dual functions in receptor binding and destruction, strictly relies on TMPRSS2 for activation on the cell membrane surface (72), exemplifying viral adaptive evolution to host systems. Moreover, secretory protease networks [e.g., plasmin (73, 74), kallikrein (KLK) (75, 76), and KLK12 (77)] can promote the spread of avian influenza viruses by specifically cleaving HA subtypes in the extracellular environment (such as respiratory secretions). Notably, TMPRSS13 plays a unique role in activating highly pathogenic avian influenza virus (HPAIV, e.g., H5N1/H7N9 subtypes) through its broad-spectrum cleavage capability (processing both monobasic and polybasic sites) and calcium-independent catalytic activity (78). Tryptase Clara and tryptase TL2 specifically recognize the consensus cleavage motif Gln (Glu)-X-Arg in influenza A and Sendai viruses to activate the viruses in the extracellular microenvironment of respiratory epithelial cells (79, 80). Conversely, some viruses (e.g., H1N1 subtypes) utilize endosomal proteases to accomplish HA cleavage after endocytosis (81).

#### 3.1.2 Multi-layered host protease regulatory networks orchestrate coronavirus invasion

Coronavirus entry is governed by the spatiotemporal coordinated activation of the spike (S) glycoprotein through host protease interplay. In SARS-CoV-2, priming cleavage at the S1/S2 junction (multibasic PRRAR motif) by furin and TMPRSS2 within the Golgi apparatus enhances virion maturation and infectivity (18, 19, 82, 83). Subsequent activation diverges into dual pathways. At the plasma membrane surface, TMPRSS2-mediated cleavage of the S2’ site exposes the fusion peptide to drive immediate virus-host membrane fusion (20, 21, 84). Endocytosed virions rely on endosomal CTSL for S protein processing (85–87) (**Figure 2B**). Notably, host protease-mediated viral entry exhibits significant bidirectionality. Coagulation factor Xa (FXa) can inhibit viral entry and infection by cleaving specific domains of the S protein, thereby blocking its binding to the ACE2 receptor (88); however, it paradoxically enhances membrane fusion efficiency through S1/S2 or S2’ cleavage in thrombotic microenvironments alongside thrombin (89). Additionally, porcine epidemic diarrhea virus (PEDV) enters cells through clathrin-mediated endocytosis in synergy with serine proteases (90), suggesting evolutionarily conserved strategies among different coronaviruses in utilizing host factors.

Host proteases exhibit functional divergence in viral invasion. TMPRSS2 and TMPRSS13 play central roles in both virus-cell fusion and subsequent cell-cell fusion stages. In contrast, HAT and differentially expressed in squamous cell carcinoma gene 1 (DESC1) show significantly weaker activation efficiency in these two fusion processes (91). TMPRSS13 has been shown to specifically promote the membrane fusion of swine acute diarrhea syndrome coronavirus (SADS-CoV) (92), suggesting the potential regulatory properties of TTSP members in determining viral host range. Metalloproteases enhance viral attachment by cleaving coronavirus spikes and ACE2 receptors, while ADAM17 facilitates viral endocytosis and is associated with inflammatory damage by mediating ACE2 shedding (93–97). Evolutionary analyses reveal that the E484 mutation enables SARS-CoV-2 to acquire cross-binding capacity with the MERS-CoV receptor dipeptidyl peptidase 4 (DPP4, also known as CD26) (98, 99), a receptor plasticity potentially attributable to furin-mediated optimization of spike protein conformation. Studies indicate that the binding of DPP4 receptors to MERS-CoV and the infection process are species-dependent. Differences in glycosylation patterns of mouse DPP4 restrict viral infection, whereas DPP4 receptors from bats, camels, and humans can support efficient viral infection (100). Notably, TMPRSS2 can also act as a receptor to bind the RBD of the human coronavirus HKU1 spike protein, inducing its conformational changes to trigger fusion (101–103), highlighting the critical role of TTSPs in cross-species transmission.

#### 3.1.3 Host protease utilization strategies in other viral families

**Figure 3:**
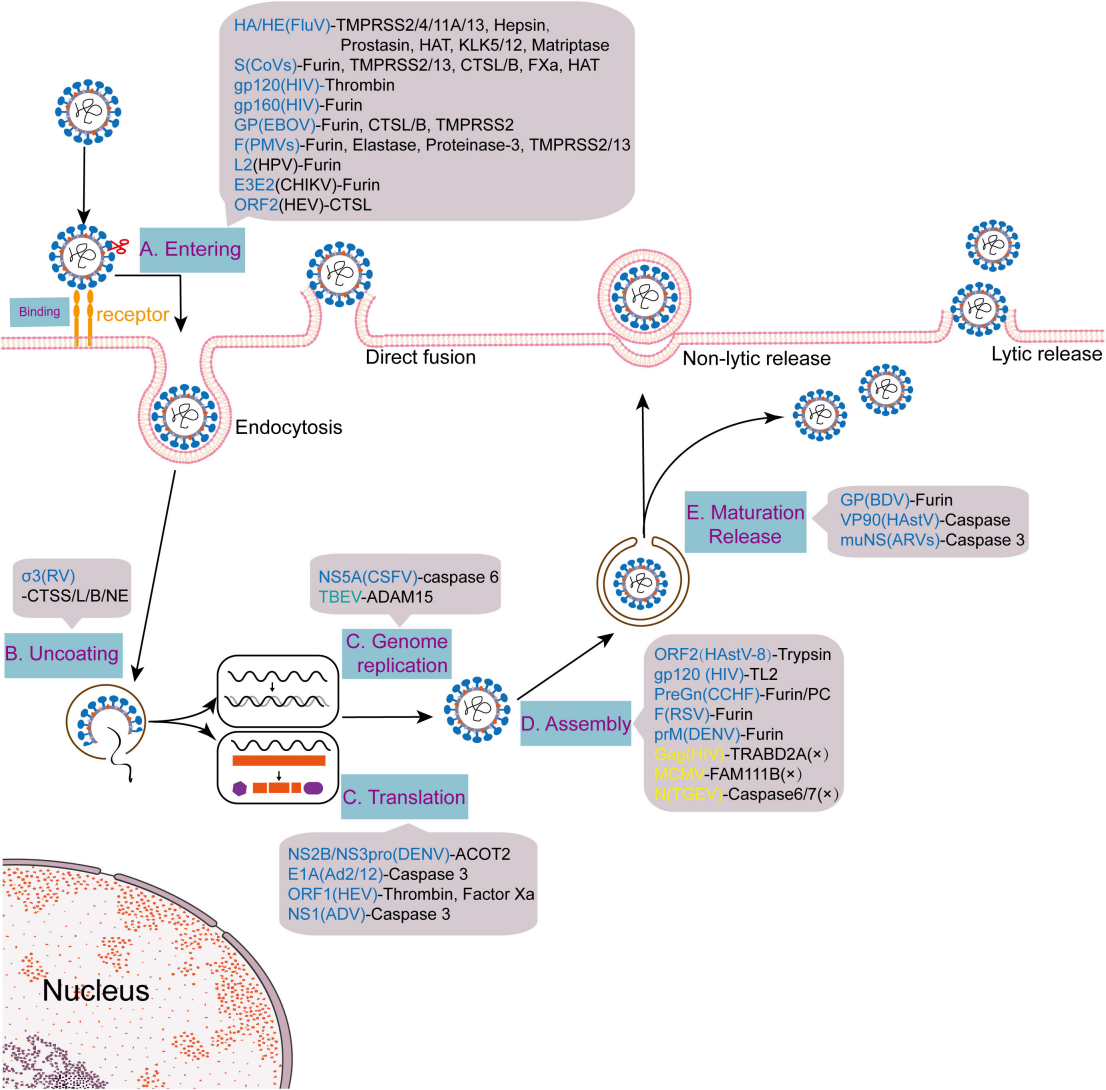
Multinodal regulation of viral life cycles by host proteases. Viruses such as influenza and SARS-CoV-2 hijack host proteases (e.g., TMPRSS2, furin) during the stages of entry, replication, assembly, and release, achieving infection through a multi-node regulatory pattern.

The utilization of host proteases represents a universal strategy for both enveloped and non-enveloped viruses during invasion (**Figure 3**). Among enveloped viruses, furin, as the core enzyme mediating the cleavage of HIV gp160, recognizes the conserved R-X-K/R-R motif in its sequence to cleave gp160 into gp120 and gp41, thereby activating viral invasion capability (12); meanwhile, thrombin enhances virus-induced cell fusion by activating HIV gp120 (104). During the initiation stage of cell fusion in placental development, the human endogenous retroviruses (HERVs) envelope protein Syncytin-1 similarly relies on furin cleavage to activate its fusion function, while Syncytin-2 maintains fusion activity via processing by the proprotein convertase subtilisin/kexin type 7 (PCSK7) (105). Filoviruses, such as EBOV, employ a proteolytic cascade activation strategy for their glycoprotein (GP). Furin mediates initial cleavage of the GP precursor in the secretory pathway, and endosomal CTSB/CTSL further trim the GP1 subunit to expose the human endosomal receptor Niemann-Pick C1 (NPC1) receptor-binding domain (14–17). Studies have confirmed that TMPRSS2 and CTSL can form a redundant mechanism to compensate for furin functional defects (106), highlighting the complexity of host protease networks. The activation of the fusion (F) protein of paramyxoviruses universally depends on host proteases. Respiratory syncytial virus (RSV) requires elastase and proteinase 3 for F protein cleavage (107), whereas human parainfluenza virus 3 (HPIV3) and mumps virus (MuV) utilize trypsin-like proteases or furin (108, 109). In-depth research reveals that TMPRSS2 and TMPRSS13 in the lung epithelium can directly cleave the HPIV3 F protein, regulating the release efficiency of infectious virions (108). Notably, furin cleavage sites exhibit cross-species conservation within the *Paramyxoviridae* family. The F proteins of HPIV3, HPIV5, virulent Newcastle disease virus (NDV) strains, measles virus (MV), and RSV all contain such sites (110–115), implying their universal value as key molecular switches. Structural conservation extends to fusion mechanisms. The post-fusion core conformations of enveloped viral fusion proteins—including SARS S, murine hepatitis virus (MHV) S, EBOV GP2, influenza virus HA2, HIV gp41, and paramyxovirus F2—exhibit striking homology (116), revealing deep evolutionary convergence across viral families. This conservation reflects a shared evolutionary strategy, where host protease activation (e.g., furin cleavage, cathepsin trimming) serves as a molecular switch that triggers conformational rearrangements from metastable pre-fusion states to stable post-fusion cores, ensuring spatiotemporally regulated membrane fusion across viral families.

Among non-enveloped viruses, HPV relies on furin-mediated cleavage of the minor capsid protein L2. The released C-terminal peptide (L2CT) not only mediates viral genome escape from endosomes but also recruits the nuclear transport factor karyopherin alpha2 (KPNA2) to guide DNA across the nuclear membrane barrier (13, 117). While L2 cleavage-deficient mutants do not affect viral attachment and endocytosis, they result in complete loss of infectivity due to failed nuclear import, indicating that furin-mediated cleavage of L2 is a critical rate-limiting step in the HPV infection cycle (118). Within the *Reoviridae* family, cleavage of the rotavirus VP4 spike protein by trypsin significantly enhances its membrane fusion capacity (119). For reoviruses, the σ3 capsid protein requires cleavage by cathepsin S (CTSS) or CTSL/B to facilitate viral uncoating and genome release (120, 121). Additionally, neutrophil elastase can promote rotavirus uncoating and infection in U937 promonocytes, substituting for CTSL to mediate a non-canonical infection pathway (122). Enteroviruses may be inactivated through conformational changes induced by serine proteases, such as subtilisin A, via capsid binding or direct cleavage, causing viral disintegration (123). Notably, polyomavirus SV40 employs a distinct strategy in quiescent cells. It activates host caspase-6 to cleave nuclear lamin A/C, inducing transient nuclear membrane deformation and dephosphorylation. This process establishes a locally softened “nuclear membrane window” to facilitate direct transport of the viral genome from the endoplasmic reticulum into the nucleus (124). This finding reveals a novel pathway by which viruses utilize host proteases to remodel the nuclear physical barriers. Similarly, the parvovirus minute virus of mice (MVM) induces caspase-3-mediated cleavage of the nuclear lamina to form physical pores, promoting capsid nuclear entry (125). Together, these findings highlight the innovative evolutionary adaptations of non-enveloped viruses in the mechanisms of nuclear membrane traversal.

Alphaviruses, such as chikungunya virus (CHIKV), rely on furin-mediated processing of their envelope protein precursor E3E2 to form functional E2-E1 heterodimers. The receptor-binding activity of these heterodimers, together with low pH-induced conformational instability, collectively drives the viral membrane fusion process. Geographic isolation has driven the evolutionary divergence of protease utilization. African strains specifically depend on membrane-bound cilia proteases and PC5B for cleavage of E3E2 at the HRQRR^64^/ST site, whereas Asian strains achieve cleavage at the RRQRR^64^/SI site via membrane-bound/soluble cilia proteases, PC5A, PC5B, and PACE4. Notably, PC7 and SKI-1 lack cleavage activity against both strain types (126), reflecting the adaptive evolution of viruses to regional host microenvironments. Additionally, hepatitis E virus (HEV) entry into hepatocytes depends on CTSL-mediated processing of viral particles and cleavage of the glycosylated ORF2 protein (127), confirming the universal role of cysteine proteases in the invasion of enveloped viruses.

### 3.2 Dynamic regulatory mechanisms of host proteases in viral replication and assembly

Host proteases precisely regulate viral replication and assembly through specific cleavage events, exhibiting multidimensional coordination and dynamic evolutionary characteristics. During the viral replication phase, host proteases play a central role in regulating key processes, such as the activation of viral precursor proteins and the formation of replication complexes (**Figure 3**). For instance, in RNA viruses, the dengue virus NS3 serine protease requires cooperation with the host serine protease acyl-CoA thioesterase 2 (ACOT2) to cleave polyproteins for functional replication complex formation (128). HEV initiates genome replication through thrombin-mediated cleavage at conserved sites of the ORF1 polyprotein (129). The caspase-6 cleavage motif (DTTD/272) in the non-structural protein NS5A of classical swine fever virus (CSFV) further confirms its regulatory role in viral replication (130). In DNA virus systems, caspase-mediated cleavage events exhibit bidirectional regulation of viral replication. Cleavage of adenovirus 2/12 (Ad2/12) early region 1A (E1A) protein by caspase-3 results in the loss of transcriptional activation function, impairing the transcriptional program necessary for efficient replication (27). In contrast, removal of the nuclear localization sequence from the NS1 protein of Aleutian mink disease parvovirus (AMDV) by caspase-3 promotes the cytoplasmic transport of ribonucleoprotein complexes, facilitating replication-related processes (131). These findings reveal the multi-target regulatory characteristics of host proteases in the viral replication process.

During virus assembly, host proteases primarily exert precise regulation by mediating the modification and maturation of viral structural proteins. The nucleocapsid protein (N) of transmissible gastroenteritis virus (TGEV) and IAV loses its genome-binding capacity after caspase-6/7 cleavage, resulting in a dramatic decrease in infectious virus particle yield (132, 133); the human astrovirus type 8 (HAstV-8) ORF2 polyprotein is specifically cleaved by trypsin to generate functional fragments that participate in capsid assembly and replication, respectively (134). Notably, protease processing strategies are viral species-specific. HIV-1 promotes the exposure of the gp41 fusion domain and the correct assembly of Env proteins through TL2 serine protease-mediated cleavage of the gp120 V3 loop (79). While furin/PC protease processing of the Crimean-Congo hemorrhagic fever (CCHF) glycoprotein precursor produces the GP38 glycoprotein, which may optimize the viral assembly environment through membrane remodeling mechanisms (135, 136). Furin cleavage of the RSV F protein is not essential for its transport but can significantly enhance viral particle assembly efficiency (137), highlighting their precise regulation of viral morphogenesis. In addition, caspase-3 mediates the nucleocytoplasmic transport of the influenza virus ribonucleoprotein complex (RNP), and its inhibition leads to RNP retention in the nucleus and triggers assembly defects. The enzyme cleaves the nuclear lamina protein Lamin A/C via a non-apoptotic pathway, remodels the nuclear membrane structure to facilitate RNP transport to the cytoplasm, and provides key components for viral particle assembly (138), further confirming the multifaceted regulatory mechanism of host proteases in viral morphogenesis.

The host-virus interaction network at the protease level is characterized by dynamic interplay and coevolution. Host factors, such as TRAB domain-containing protein 2A (TRABD2A), can inhibit viral assembly by degrading the Gag protein of HIV-1 (139), while the primate-specific restriction factor FAM111B inhibits the replication of mouse cytomegalovirus (MCMV) in human cells by enriching in viral replication regions—a restriction stemming from the fact that MCMV has not evolved strategies against family with sequence similarity 111 member B (FAM111B) in its natural hosts (rodents) (140). Studies on cross-species transmission reveal that viruses can actively utilize host proteases to break through barriers. The conserved cleavage of HEV pORF1 by thrombin and FXa is a key basis for its ability to cross host boundaries (141). Tick-borne encephalitis virus (TBEV) reorganizes the membrane system by relocating host ADAM15 protease to its replication region, thereby optimizing its own replication environment (142). Such adaptive strategies frequently drive systematic mutations in the cleavage sites of viral proteases.

### 3.3 Diverse regulatory mechanisms of host proteases in viral release and transmission

Host proteases profoundly enhance viral particle release and transmission efficiency through precise regulation of viral maturation and microenvironment remodeling (**Figure 3**). The core function of furin is evolutionarily conserved across viral families during terminal maturation. For example, furin-mediated cleavage of HIV gp160 enhances viral particle infectivity and induces conformational rearrangements to evade neutralizing antibodies (28, 29); flaviviruses (e.g., DENV and ZIKV) require furin cleavage of prM to M protein for mature particle formation, though incompletely cleaved immature particles retain infectivity via non-canonical entry pathways that exacerbate disease severity (30). While Borna disease virus (BDV) glycoprotein (GP, encoded by ORF-IV) strictly depends on site-specific furin cleavage at Arg249 to maintain bioactivity (143). These collective mechanisms underscore how proteolytic processing fine-tunes viral dissemination strategies across diverse species.

Viral cell-to-cell transmission involves a more extensive proteolytic regulatory network. Although furin cleavage of pseudorabies virus glycoprotein B (gB) is dispensable for *in vitro* viral replication, it remains critical for mediating membrane fusion and syncytium formation (144, 145). MMP-9 significantly enhances HIV cell-to-cell transmission by degrading extracellular matrix components (47). Additionally, caspase family members play pivotal roles in facilitating viral release. They cleave the human astrovirus capsid precursor VP90 to form mature capsids, thereby promoting viral release (146); while they dissolve cytoplasmic inclusion bodies maintained by Avian reoviruses (ARVs) mμNS protein to expel mature particles (147). Collectively, these diverse mechanisms highlight the intricate deep coevolutionary relationship between viruses and the host protease system.

### 3.4 Host protease regulatory networks in immune response and evasion

Host proteases construct multidimensional regulatory networks spanning molecular cleavage to systemic immunity during viral immune responses and evasion strategies (**Table 2**). Viruses achieve immune evasion by hijacking the protease activity. For instance, influenza viruses employ TMPRSS2 not only to enhance viral membrane fusion efficiency, but also to promote vascular permeability by activating the “influenza virus-cytokine-trypsin” cycle. The upregulated trypsin and pro-inflammatory cytokines exacerbate tissue destruction and immune suppression, enabling the virus to evade immune clearance and continue to replicate (148). Similarly, cathepsin G (CTSG) recruits monocytes/macrophages to inflammatory sites during HIV-1 infection and heightens their viral susceptibility, establishing a positive feedback loop (25). Conversely, the host has evolved protease-based antiviral defenses—neutrophil serine proteases (NE/PR3/CTSG) directly cleave the SARS-CoV-2 spike protein to block viral entry (149), while myeloid-specific serine proteases interfere with NF-κB activation by processing its p65 subunit, thereby inhibiting critical HIV replication processes (24). This bidirectional protease warfare underscores the evolutionary arms race at the host-pathogen interface.

**Table 2:** Immune regulatory functions of host proteases in viral infections.

Protease	Immunological mechanism	Effect direction	Related viruses	Targeting significance	References
Neutrophil elastase	Cleave viral spike proteins to block cellular entry	Defense	SARS-CoV-2	Block early infection	(149)
MASP-2	Activate complement system causing cytokine storm	Damage	SARS-CoV-2	Mitigate severe organ injury	(167)
MMP-9	Suppress IFN signaling + Promote fibrosis	Bidirectional	HBV, RSV	Anti-fibrotic therapy	(46, 162, 163)
	Enhance vascular endothelial permeability	Damage	DENV	Reduce hemorrhagic fever	(160)
	Disrupt the blood-brain barrier	Damage	MAV-1	Prevent encephalitis	(164)
MMP-8	Degrade tight junction proteins to disrupt the blood-brain barrier	Damage	RABV	Protect the neural barrier	(170)
MMP-10	Upregulate NF-κB/JAK-STAT dual pathways and degrade extracellular matrix	Damage	RSV	Block airway remodeling	(171)
CTSG	Recruit infection-promoting immune cells	Evasion	HIV-1	Prevent “infectious niche” formation	(25)
ADAM17	Mediate ACE2 shedding to promote viral endocytosis, associated with inflammatory damage	Damage	SARS-CoV-2	Alleviate acute lung injury	(95–97)
	Key attachment factor	Defense	BVDV/CSFV	Block viral cross-species transmission	(168)
	Stabilize immune receptors and inhibit antiviral responses	Evasion	HCMV	Block immune evasion	(169)
TMPRSS2	Induce cytokine storm cycle	Damage	Influenza virus	Prevent multi-organ failure	(148)
TMPRSS11D	Activate prothrombin, triggering acute fibrin deposition	Damage	SARS-CoV-2	Alleviate damage	(159)
MMP-3	Nuclear translocation enhances NF-κB signaling, promoting antiviral cytokine secretion	Defense	VSV, H1N1, DENV	Broad-spectrum immune enhancer	(23, 166)
FAM111B	Enrich in viral replication regions, restricting MCMV replication in human cells	Defense	MCMV	Target for cross-species transmission barrier	(140)
HtrA2/Omi	Cleavage of viral proteins triggers apoptosis, restricting viral spread	Defense	CMV	Pro-apoptotic antiviral strategy	(26)

“-” represents “unidentified.” BVDV, Bovine Viral Diarrhea Virus; RABV, Rabies Virus.

The precise regulation of host protease activity by host factors constitutes a critical mechanism in antiviral defense. Interferon-induced guanylate binding proteins (GBPs, such as GBP2 and GBP5) inhibit furin and PCSK family activity, impeding the maturation of viral envelope glycoprotein precursors, including HIV-1 gp160, and significantly reducing the infectivity of various viruses, including HIV-1, ZIKV, MV, and IAV (150). Alpha-soluble NSF attachment protein (α-SNAP) binds to the P-domain of furin, inhibiting cleavage of the SARS-CoV-2 spike protein and other furin-dependent viral glycoproteins (151). Members of the serine protease inhibitor superfamily (SERPIN) also play essential roles, alpha (1)-antitrypsin (A1AT) inhibits TMPRSS2 and ADAM17, blocking SARS-CoV-2 spike protein activation and ACE2 shedding (152–154), while plasminogen activator inhibitor-1 (PAI-1) regulates fibrinolysis and additionally inhibits the enzymatic activity of proteases including FXIIa and TMPRSS2 (155). Furthermore, cystatin C (CST3) competitively inhibits the activity of CTSB/CTSL through its N-terminal region, while its dimer form enhances CTSB activity by binding to a structure-specific allosteric pocket of CTSB (156). Interleukin-1β (IL-1β) activates ADAM17 through phosphorylation (157). Collectively, these regulatory networks of host factors over protease activities profoundly influence viral infection processes.

The functions of host proteases frequently exhibit tissue- and cell-compartment specificity, and profoundly influence systemic immune responses. In renal cells, SARS-CoV-2 evades the inhibitory effect of the host restriction factor nuclear coactivator 7 (NCOA7) via a TMPRSS2-mediated non-endosomal pathway (158). Conversely, when SARS-CoV-2 infects human bronchial epithelial cells, it induces ST14/TMPRSS11D to activate prothrombin, triggering acute fibrin deposition (159). At the systemic level, SARS-CoV-2 activates the NETs-PAD-4 pathway to induce lung epithelial cell death (22), whereas dengue virus is directly linked to imbalances in the coagulation-fibrinolytic system through a metalloproteinase-mediated vascular leakage mechanism (160). Moreover, the degree of coagulation and fibrinolytic activation induced by it is positively correlated with disease severity (161).

The matrix metalloproteinase (MMP) family exhibits complex and differentiated functions in viral immunomodulation. In HBV infection, MMP-9 promotes viral replication and hepatic fibrosis by inhibiting interferon signaling (46). RSV infection efficiently stimulates MMP-9 expression *in vivo* and *in vitro* (162), while disruption of the MMP-9/TIMP-1 balance drives airway remodeling—a key pathogenic feature of chronic pulmonary fibrosis (163). In a neuroinvasive model, mouse adenovirus (MAV-1) activates microglial MMP-2/MMP-9 to disrupt the blood-brain barrier, representing a critical pathological mechanism underlying encephalitis development (164). Studies on MHV infection further revealed that increased viral replication during lethal infection is closely associated with significantly elevated expression levels of MMPs, TIMPs, and chemokine genes (165). Conversely, MMP-3 exerts broad-spectrum antiviral activity against vesicular stomatitis virus (VSV), H1N1, and HSV-1 through NF-κB signaling potentiation via nuclear translocation, while simultaneously enhancing anti-dengue immune responses (23, 166).

Complement system regulation represents another critical battleground for viral immune evasion. Aberrant interactions between the mannose-binding lectin-associated serine protease 2 (MASP-2) and the SARS-CoV-2 N protein drive complement hyperactivation, fueling cytokine storms and multiorgan damage—a mechanism particularly prominent in severe COVID-19 (167). Conversely, the high-temperature requirement protein A2 (HtrA2/Omi) effectively limits cytomegalovirus (CMV) spread by triggering apoptotic pathways through cleavage of key viral or host proteins (26). Long-term host-virus coevolution has forged dynamic equilibria in proteolytic cleavage sites. ADAM17, a pivotal immunoregulatory node, plays essential roles in host defense against pestiviruses (168), while being targeted by cytomegaloviruses to remodel the cell surface proteome (169). Neuroinvasive viruses exploit MMP-8 to degrade tight junctions in the blood-brain barrier (170), whereas RSV infection induces MMP-10 expression in nasal epithelial cells and modulates the immune microenvironment through NF-κB/JAK-STAT crosstalk (171). These mechanisms collectively demonstrate how viruses achieve immune microenvironment remodeling via multidimensional regulation of proteolytic networks.

## 4 Antiviral intervention strategies targeting host proteases: from single-target inhibition to multidimensional synergistic regulation

Compared with the drug resistance challenges posed by high-frequency mutations in viral genomes, host proteases, due to the high genetic stability of their encoding genes, emerge as highly attractive targets for developing host-directed antiviral drugs (HADs). This approach significantly mitigates risks of viral escape mutations. Furthermore, inhibitors targeting host proteases generally exhibit broad-spectrum antiviral potential, providing a feasible approach to combat multiple viral infections. With the deepening understanding of the viral infection complexity and immune evasion mechanisms, intervention strategies targeting these critical regulatory nodes in the viral life cycle are dynamically evolving from traditional single-target inhibition to multidimensional synergistic regulation.

### 4.1 Continuous advancement in single-target inhibition research

**Figure 4:**
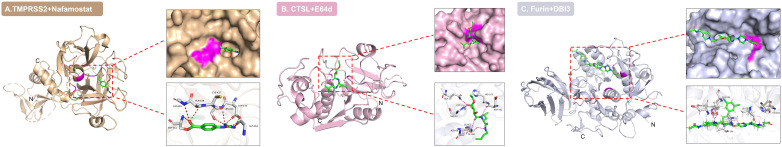
Active site binding modes of host protease-inhibitor complexes. Three-dimensional structures of **(A)** TMPRSS2-nafamostat (PDB: 7MEQ), **(B)** CTSL-E64d (PDB: 8HET), and **(C)** furin in complex with dichlorophenylpyridine-based inhibitor 3 (DBI3, PDB: 7QXY) are shown. Inhibitors (green) targetedly insert into the active sites of proteases, forming key interactions with catalytic residues and surrounding amino acids (e.g., Ser441 in TMPRSS2, Cys25 in CTSL, Asp154 in furin) via hydrogen bonds and hydrophobic interactions. The catalytic centers are highlighted in magenta.

Currently, research on single-target inhibitors targeting key host proteases continues to deepen. Small-molecule inhibitors remain the primary focus due to their favorable drugability and high maturity in development. In the field of targeting transmembrane serine proteases, the TMPRSS2 inhibitor nafamostat (**Figure 4A**) blocks 93% of SARS-CoV-2 plasma membrane invasion but exhibits limited inhibitory effects on TMPRSS4-dependent MERS-CoV (20, 172). The new-generation inhibitor N-0385, with low nanomolar potency, effectively inhibits the invasion of variants, including Omicron (173, 174). While nafamostat mesylate can reduce viral load in murine lungs (175), its clinical efficacy is limited by rapid cleavage and inactivation by TMPRSS11D (176). Other broad-spectrum serine protease inhibitors, such as 4-(2-aminoethyl) benzenesulfonyl fluoride (AEBSF) and N-alpha-tosyl-L-phenylalanyl chloromethyl ketone (TPCK), can inhibit RSV infection, among which AEBSF acts primarily at the early stage of viral entry (177). Structurally optimized HAT serine protease inhibitors (e.g., compound 15, *K_i_* = 15 nM) enhance selectivity through novaricine modification, thereby effectively inhibiting influenza virus replication (178). Additionally, basic phenylalanine analogs reduce the titers of West Nile virus (WNV) and DENV by 10,000-fold via plasmin inhibition (179).

Classical cysteine protease inhibitors targeting CTSL demonstrate broad-spectrum antiviral efficacy. *In vitro* models, inhibitors E64d (**Figure 4B**) and Z-FY-CHO effectively suppress SARS-CoV-2 pseudovirus infection (20, 83, 180, 181), while the broad-spectrum inhibitor K11777 exhibits potent inhibitory activity against HEV, with an EC_50_ of approximately 0.02 nM (127). *In vivo*, rotavirus capsid disassembly strictly depends on CTSL, and treatment with the inhibitor Z-Phe-Tyr(t-Bu)-diazomethyl ketone causes drastic viral yield reduction (121). Additionally, amantadine, an anti-influenza drug, blocks SARS-CoV-2 infection by inhibiting CTSL activity (87), while the natural product gallinamide A and its analogs also exhibit potent antiviral activity (182).

Furin inhibitor development has achieved significant advances across multiple fronts. The competitive inhibitor dec-RVKR-cmk can block F protein cleavage and viral budding of RSV (137), while exerting anti-SARS-CoV-2 activity by inhibiting spike protein cleavage and syncytium formation (183), though it is ineffective against filoviruses (184). This compound additionally suppresses flavivirus release without affecting RNA replication (185). In terms of allosteric inhibitors, cypermethrin exhibits broad-spectrum activity against drug-resistant SARS-CoV-2 by binding to a novel allosteric pocket (186), while the reversible inhibitor MI-1851 reduces viral load by 190-fold (83, 187). It is worth noting that protease inhibitors designed based on the (3,5-dichlorophenyl) pyridine skeleton (**Figure 4C**) exhibit high potency and antiviral activity against SARS-CoV-2 at the cellular level (188), providing new insights for broad-spectrum antiviral treatment. The natural product luteolin inhibits furin in a non-competitive manner and significantly reduces DENV viremia *in vivo* (189). Emerging approaches explore novel paradigms. Targeting the off-state of furin opens new avenues for the design of selective inhibitors (190). Decanoyl-RVKR-CMK effectively blocks E3E2 precursor cleavage by CHIKV (30). And furin conformation provides new opportunities for structure-based drug discovery (186), demonstrating the feasibility of developing customized inhibitors for specific viruses.

In addition to small-molecule inhibitors, peptide-based compounds, endogenous regulatory factors, and biologics serve as vital complements to single-target inhibition strategies due to their targeting specificity and biocompatibility advantages. In peptide inhibitor research, polyarginine repeat sequences function as competitive inhibitors of furin substrate cleavage, effectively inhibiting HIV infection by blockade of gp160 protein processing (191). Peptides P9 and P9R significantly reduce SARS-CoV-2 viral load in hamster models by impairing CTSL activity (192, 193). The TMPRSS13 peptidomimetic inhibitor N-0430 blocks SARS-CoV-2 pseudovirus entry (194), while the caspase-6 inhibitor Z-VEID-FMK alleviates pathological damage in SARS-CoV-2 and MERS-CoV animal models (195). Recombinant applications of endogenous inhibitors demonstrate substantial progress. Interferon-induced GBP5 protein inhibits furin activity, markedly reducing infectivity of multiple pathogens, including HIV-1, Zika virus (ZIKV), MV, and IAV (150). Serpin family B member 8 (Serpin B8, also known as PI8 and CAP2) binds to and inhibits the proprotein convertase furin (28). Furthermore, α-SNAP suppresses furin-dependent viral glycoprotein cleavage through binding to the furin P-domain (149). In the field of biologics, nanobodies exhibit exceptional advantages due to their high specificity. The anti-TMPRSS2 nanobodies inhibit the enzymatic activity of TMPRSS2 and hinder HKU1 pseudovirus entry using S441A TMPRSS2 (101). Dromedary heavy-chain-derived nanobodies specifically inhibit the catalytic activity of furin, blocking its cleavage of two critical substrates, transforming growth factor beta (TGFβ) and glypican-3 (GPC3) (196).

### 4.2 Translational breakthroughs in inhibitor synergy strategies

Multi-target synergistic strategies are overcoming the limitations of single-inhibitor therapies. Clinical studies have shown that the TMPRSS2 inhibitor N-0385, when combined with the antiviral drugs remdesivir or nirmatrelvir, exhibits broad-spectrum synergistic activity against Omicron subvariants (174). In chronic hepatitis B treatment, entecavir coupled with furin inhibitors concurrently suppresses viral replication and hepatitis B e antigen (HBeAg) secretion (197). Spironolactone enhances antiviral effects by antagonizing TMPRSS2/ADAM17 to reduce soluble ACE2, synergizing with DPP-4 inhibitors to improve clinical outcomes in COVID-19 patients (198). Additionally, xanthan gum combined with camostat significantly enhances anti-influenza virus potency (199). Mechanistic studies further confirmed that non-toxic furin inhibitors combined with TMPRSS2 inhibitors block 95% of lung cell infections (200), demonstrating the translational potential of inhibitor synergy strategies in multistep blockade of viral invasion.

### 4.3 Innovative waves in multi-target drug development

Dual- and multi-target therapeutics are spearheading novel antiviral strategies. Compound BAPA exhibits an EC_50_ of 0.3 μM against H1N1 by inhibiting HAT/TMPRSS2 (201). The tri-targeting peptidomimetic MM3122 simultaneously inhibits TMPRSS2, matriptase, and hepsin, maintains sub-nanomolar potency against the SARS-CoV-2 EG.5.1 variant, and significantly attenuates pulmonary edema in mice (202). Delivery system innovations propel the development of bispecific compound 212-148, which simultaneously inhibits TMPRSS2 and CTSL/CTSB (203), with nanoerythrocyte carriers substantially enhancing delivery efficiency (204). Diazoxide inhibits TMPRSS2/furin (IC_50_=1.35/13.2 μM), while compound MI-1148 blocks transmission of highly pathogenic avian influenza (HPAI) and canine distemper virus by targeting PC1/3 (205). Notably, the mechanisms of action of protease inhibitors nafamostat and camostat may extend beyond TMPRSS2 inhibition itself, involving coagulation cascade-induced cleavage of spike proteins. Given the centrality of anticoagulation management in COVID-19 therapy, early intervention may provide synergistic benefits by blocking viral entry (89).

Structure-guided design has achieved pivotal breakthroughs. The α-ketoamide inhibitors 14a/14b exhibit potent broad-spectrum anti-coronaviral activity through covalent binding to CTSL and calpain-1 (CAPN1), achieving exceptional potency against SARS-CoV-2 variants (EC_50_ as low as 0.80 nM) (206). The natural product omicsynin B4 demonstrates pan-coronaviral activity against human coronavirus 229E (HCoV-229E), human coronavirus OC43 (HCoV-OC43), and SARS-CoV-2 prototype/variants by dual blockade of CTSL/TMPRSS2 (207). At the level of respiratory protease regulation, influenza HA activation mediated by human eosinophils and DESC1 (but not TMPRSS11A) is specifically inhibited by hepatocyte growth factor activator inhibitor 1 (HAI-1) (66). The endogenous regulator serine protease inhibitor Kazal‐type 6 (SPINK6) inhibits HAT/KLK5 to restrict influenza virion maturation (208), while dichlorobiphenyl-containing matriptase inhibitors achieve ultrahigh potency (*K_i_* < 3 nM) through chemical optimization, demonstrating exceptional thrombin selectivity and concentration-dependent inhibition of H9N2 viral replication in MDCK(II) cells (209). Among matriptase/TMPRSS2 inhibitors evaluated by Gamba, D. et al., MI-463 and MI-1900 exhibit antiviral effects against H1N1/H9N2 at concentrations of 20-50 µM, suggesting that they block viral entry by inhibiting host protease-mediated cleavage (210). The oral dual-target drug olgotrelvir, which simultaneously inhibits SARS-CoV-2 M^pro^ and CTSL, has emerged as a new paradigm for clinical translation (211).

### 4.4 Translational challenges and cutting-edge strategies

Despite extensive development of host protease inhibitors demonstrating antiviral potential in preclinical models, few have successfully transitioned to clinical application. The current translational bottlenecks primarily stem from three key challenges. First, host compensatory escape—viruses can not only bypass inhibition by activating functionally redundant host proteases, such as SARS-CoV-2 switching from TMPRSS2-dependent entry to CTSL-mediated invasion pathways (85–87); they can also utilize their own encoded proteases to compensate for critical functions. For instance, HCV relies solely on its NS3/4A serine protease with NS4A as a cofactor to independently cleave viral polyproteins (212). Similarly, DENV requires its NS3 protease—an essential component for nonstructural protein hydrolysis—which functions with its own NS2B cofactor (213). Meanwhile, SARS-CoV-2 processes polyproteins pp1a and pp1ab through its M^pro^ to generate 16 mature nonstructural proteins (nsp1-nsp16), which collectively form the replication/transcription complex that provides core support for viral replication (214). Second, off-target toxicity—broad-spectrum inhibitors (e.g., camostat) inhibit TMPRSS2 while interfering with proteases involved in coagulation, inflammation, and blood pressure regulation, significantly increasing the risk of serious adverse events in clinical treatment groups (215); third, tissue delivery obstacles—small-molecule inhibitors struggle to penetrate specific compartments (e.g., inactivation in the acidic lysosomal environment, blockage by the blood-brain barrier).

It is noteworthy that multi-target synergistic strategies aimed at enhancing antiviral efficacy, such as dual-target proteolysis-targeting chimera (PROTAC) degraders, may increase off-target risks due to the expanded range of target molecules. Current research seeks breakthroughs through two precision-optimized design approaches. One leverages spatial precision by confining activity release ranges using tissue-microenvironment-responsive carriers (216), while another employs conformational precision through allosteric site engineering to selectively engage inactive states of host proteases, thereby enhancing specificity (217). These approaches pave a critical pathway for balancing synergistic potency and safety. Thus, developing novel inhibitors with high selectivity, resistance barriers, and microenvironmental adaptability has become an urgent need to address the challenges of viral evolution. Cutting-edge strategies focus on three breakthroughs. One is the exploration of allosteric inhibitory sites (e.g., targeting furin exosite-III). Another is the design of dual-target PROTAC degraders (e.g., simultaneous degradation of TMPRSS2/CTSB). The third is the development of smart responsive nanocarriers (e.g., pH-sensitive liposomes loaded with cystatin C targeting endosomes).

## 5 Conclusion

Viral infections can trigger various severe diseases, such as pneumonia, meningitis, hepatitis, and cardiovascular diseases, posing a significant threat to human health. The regulatory role of host proteases in viral infections has transcended traditional understanding. As one of the core dynamic hubs in the virus-host interaction network, these enzymes not only directly drive critical processes, including viral entry, replication, and immune evasion, but also profoundly reshape infection progression through spatiotemporal activity regulation. Viruses typically hijack host protease activity to facilitate infection, with their specificity and activity directly determining viral pathogenicity. Research has elucidated three core viral evolutionary strategies enabling cross-species transmission—hijacking tissue-enriched proteases (e.g., TMPRSS2 in respiratory epithelial cells); inducing abnormal activation of microenvironment proteases (e.g., inflammation-driven MMP-9 overexpression); and optimizing adaptability of cleavage sites (e.g., the PRRA motif in the SARS-CoV-2 S protein). These mechanisms provide molecular foundations for understanding viral pathogenicity variations. In therapeutic development, host protease targeting is transitioning from single-inhibition approaches toward multidimensional synergistic paradigms. Innovative designs, including dual-target PROTAC degraders, allosteric inhibitors, and intelligent delivery systems, mark a significant turning point in the field. However, clinical translation encounters three persistent challenges—host compensatory escape mechanisms, off-target toxicity, and delivery barriers. Future breakthroughs require a focus on space-conformation precision technologies, such as employing microenvironment-responsive carriers to restrict active compound distribution or designing selective binding to inactive states of proteases based on allosteric sites, thereby balancing efficacy with physiological safety.

Looking forward, host protease-targeted therapy progress will focus on three interconnected dimensions. At the mechanism-elucidation level, cryo-EM and molecular dynamics simulations reveal dynamic conformational changes of protease-substrate complexes (e.g., the transient intermediates formed during furin cleaves the S protein), providing atomic-resolution blueprints for allosteric inhibitor design. At the technological development level, an AI-driven multi-target degradation agent screening for multi-target degraders integrates, host proteomics and viral evolution data to predict optimal target combinations (e.g., the combined intervention of TMPRSS2 and CTSL). At the clinical translation level, it requires establishing tiered organoid-animal model evaluation systems to assess tissue-specific toxicity in human-mimetic microenvironments (e.g., the long-term consequences of prostate TMPRSS2 suppression), alongside exploring sequential therapies against viral escape. The paramount value of these advances lies not only in significantly reducing the risk of target mutation-driven drug resistance—the genetic stability of host proteases makes them an “anchor” for controlling highly variable viruses (such as HIV and DENV)—but also in providing broad-spectrum countermeasures for emerging viral outbreaks. From respiratory to neuroinvasive viruses, host protease-targeting strategies are transforming antiviral development paradigms. Realizing this vision demands deep integration of virology, structural biology, and nanomedicine. Consequently, developing novel intervention strategies targeting host proteases holds broad application prospects and significant research value in the field of antiviral therapy.

## Glossary

α-SNAP: alpha-soluble NSF attachment protein
A1AT: αlpha (1)-antitrypsin
ACOT2: acyl-CoA thioesterase 2
ADAM17: a disintegrin and metalloproteinase 17
Ad2/12: adenovirus 2/12
AEBSF: 4-(2-aminoethyl) benzenesulfonyl fluoride
AMDV: Aleutian mink disease parvovirus
ARVs: Avian reoviruses
Asp: aspartic
β-NGF: β-nerve growth factor
BDV: Borna disease virus
CAPN1: calpain-1
CCHF: Crimean-Congo hemorrhagic fever
CHIKV: chikungunya virus
CMV: cytomegalovirus
COVID-19: coronavirus disease 2019
cryo-EM: cryo-electron microscopy
CSFV: classical swine fever virus
CST3: cystatin C
CTSB: cathepsin B
CTSD: cathepsin D
CTSG: cathepsin G
CTSL: cathepsin L
CTSS: cathepsin S
DENV: dengue virus
DESC1: differentially expressed in squamous cell carcinoma gene 1
DPP4: dipeptidyl peptidase 4
E166V: Glu166Val
E1A: region 1A
EBOV: Ebola virus
ENaC: epithelial sodium channel
FAM111B: family with sequence similarity 111 member B
FluV: influenza virus
Fxa: coagulation factor Xa
gB: glycoprotein B
GBPs: guanylate binding proteins
GP: glycoprotein
GP1: glycoprotein 1
GPC3: glypican-3
H1N1: hemagglutinin 1 neuraminidase 1
HA: hemagglutinin
HADs: host-directed antiviral drugs
HAI-1: hepatocyte growth factor activator inhibitor 1
HAstV-8: human astrovirus type 8
HAT: human airway trypsin-like protease
HBeAg: hepatitis B e antigen
HBV: hepatitis B virus
HCV: hepatitis C virus
HCoV-229E: human coronavirus 229E
HCoV-HKU1: human coronavirus HKU1
HCoV-OC43: human coronavirus OC43
HE: hemagglutinin-esterase
HERVs: human endogenous retroviruses
HEV: hepatitis E virus
HIV: human immunodeficiency virus
HPIV3: human parainfluenza virus 3
HPV: human papillomavirus
ICV: Influenza C virus
IL-1β: interleukin-1β
KPNA2: karyopherin alpha2
L2: late protein 2
MASP-2: Mannose-binding lectin-associated serine protease 2
MAV-1: mouse adenovirus
MCMV: mouse cytomegalovirus
MHV: murine hepatitis virus
MMP: matrix metalloproteinase
MMP-2: matrix metalloprotease-2
MMP-9: matrix metalloprotease-9
MuV: mumps virus
MV: measles virus
MVM: minute virus of mice
NCOA7: Nuclear receptor coactivator 7
NDV: Newcastle disease virus
NPC1: the human endosomal receptor Niemann-Pick C1
PAI-1: plasminogen activator inhibitor-1
PCSK7: proprotein convertase subtilisin/kexin type 7
PEDV: porcine epidemic diarrhea virus
pro-OCN: pro-osteocalcin
PROTAC: proteolysis-targeting chimera
RBD: receptor-binding domain
RNP: ribonucleoprotein
RSV: Respiratory syncytial virus
SADS-CoV: swine acute diarrhea syndrome coronavirus
SARS-CoV-2: severe acute respiratory syndrome coronavirus 2
SERPIN: serine protease inhibitor superfamily
SPINK6: Serine protease inhibitor Kazal‐type 6
TBEV: tick-borne encephalitis virus
TGFβ: transforming growth factor beta
TGEV: transmissible gastroenteritis virus
TMPRSS: transmembrane serine protease
TPCK: N-alpha-tosyl-L-phenylalanyl chloromethyl ketone
TRABD2A: TRAB domain-containing protein 2A
TTSP: type II transmembrane serine protease
VSV: Vesicular Stomatitis Virus
WNV: West Nile virus
ZIKV: Zika virus
